# Supplementary figures and images for: Human CD29+/CD56+ myogenic progenitors display tenogenic differentiation potential and facilitate tendon regeneration
Source: eLife. 2025 May 19;13:RP98636. doi: 10.7554/eLife.98636 (PMC12088673; doi:10.7554/eLife.98636)

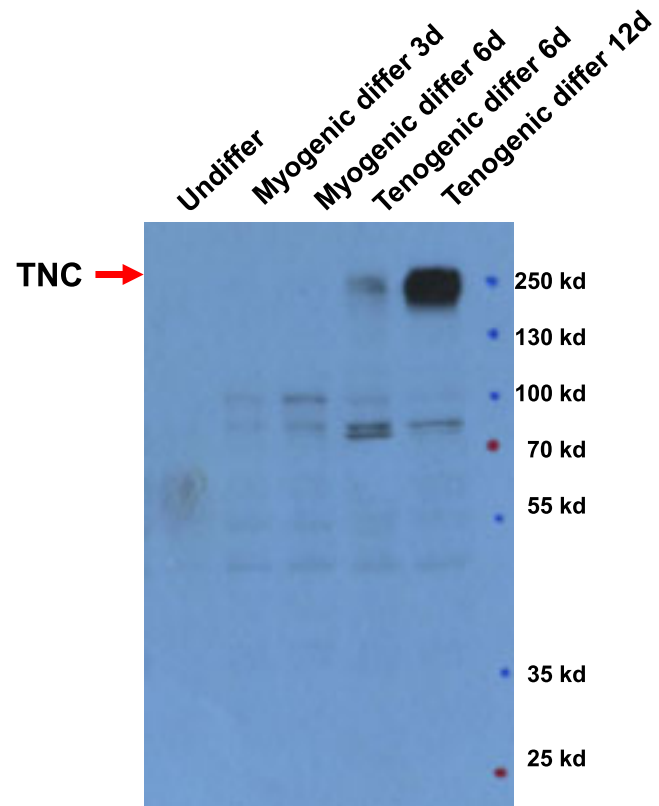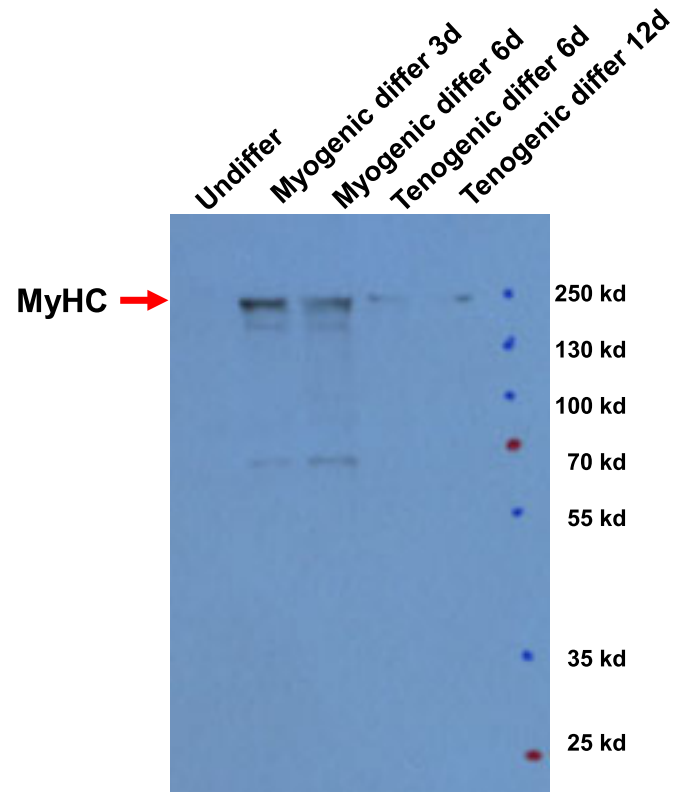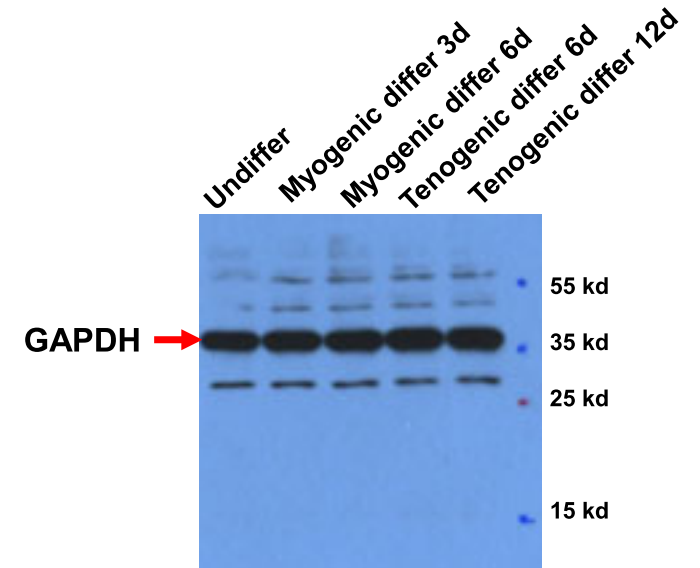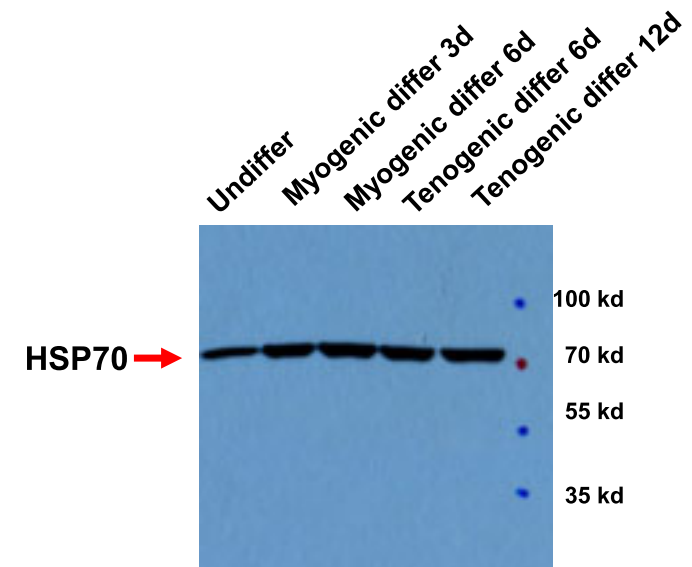

Supplement: Figure 2—figure supplement 1—source data 1. [file elife-98636-fig2-figsupp1-data1.zip › Figure 2-figure supplement 1_Source Data 1/Figure 2-figure supplement 1_Source Data 1.pdf]

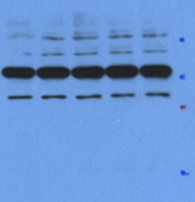

Supplement: Figure 2—figure supplement 1—source data 2. [file elife-98636-fig2-figsupp1-data2.zip › Figure 2-figure supplement 1_Source Data 2/GAPDH.png]

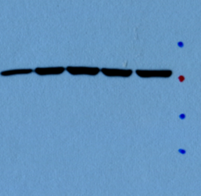

Supplement: Figure 2—figure supplement 1—source data 2. [file elife-98636-fig2-figsupp1-data2.zip › Figure 2-figure supplement 1_Source Data 2/HSP70.png]

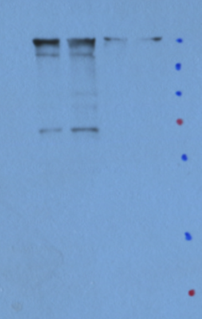

Supplement: Figure 2—figure supplement 1—source data 2. [file elife-98636-fig2-figsupp1-data2.zip › Figure 2-figure supplement 1_Source Data 2/MYHC.png]

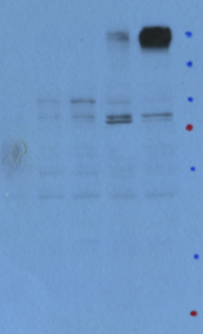

Supplement: Figure 2—figure supplement 1—source data 2. [file elife-98636-fig2-figsupp1-data2.zip › Figure 2-figure supplement 1_Source Data 2/TNC.png]

TNC

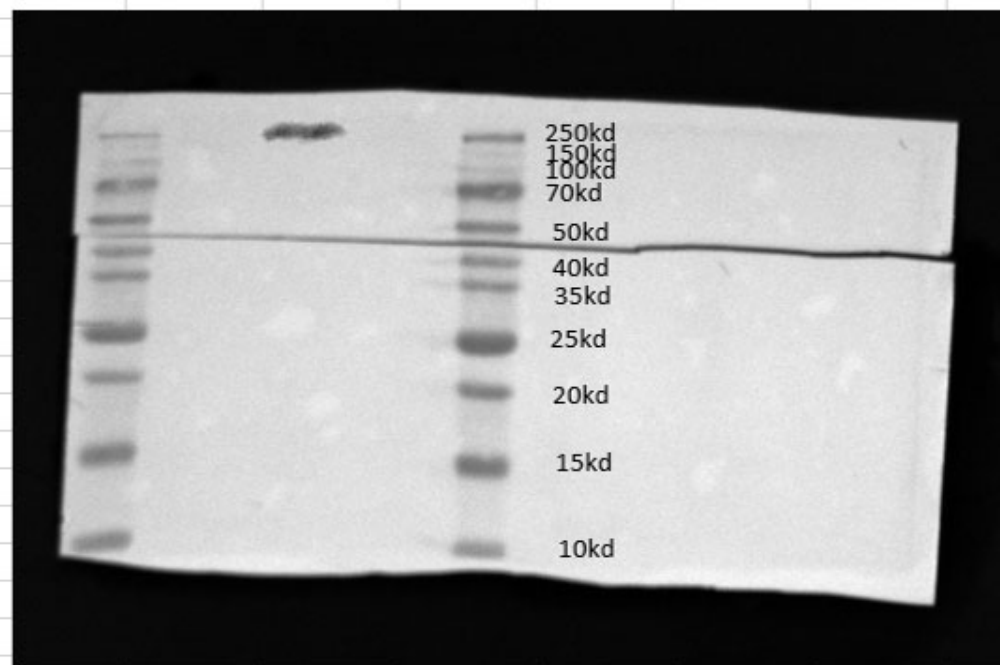

GAPDH

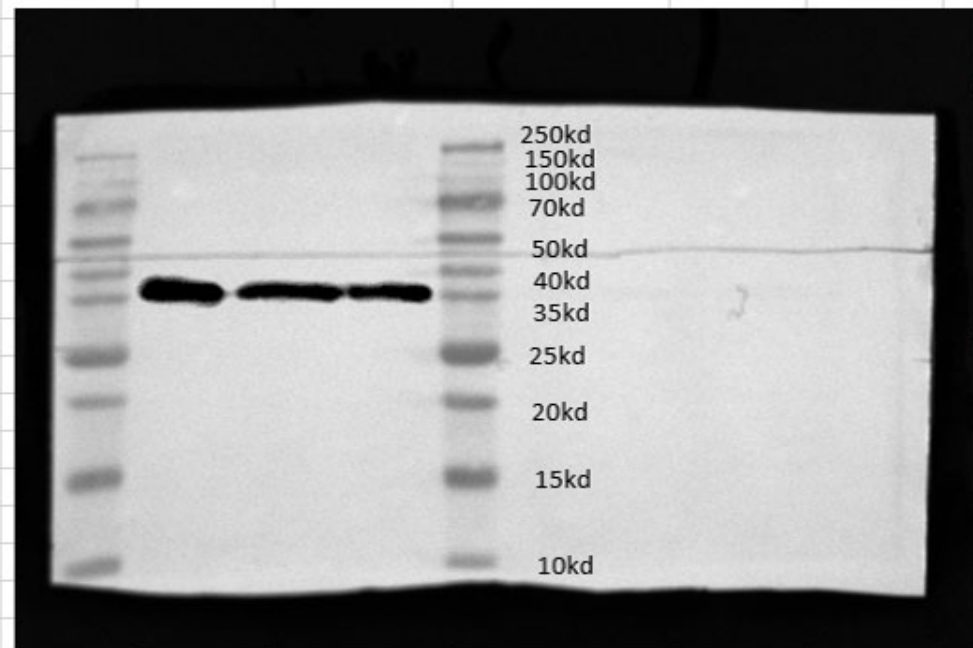

SCX

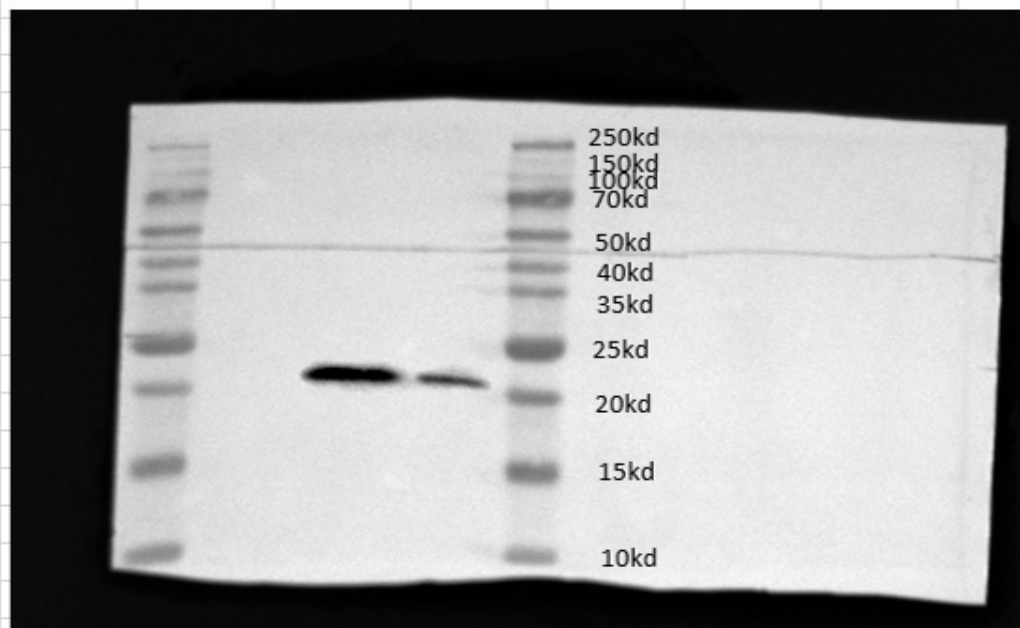

Supplement: Figure 7—source data 1. [file elife-98636-fig7-data1.zip › Figure7_Source Data 1/Figure7_Source Data 1.pdf]

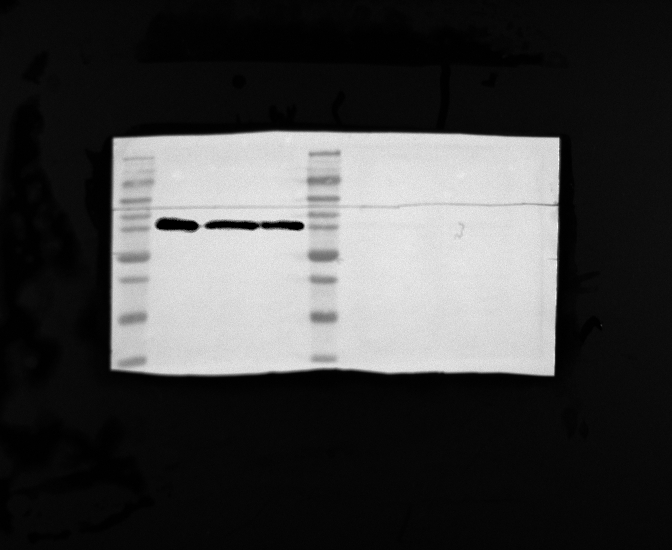

Supplement: Figure 7—source data 2. [file elife-98636-fig7-data2.zip › Figure7_Source Data 2/GAPDH.jpg]

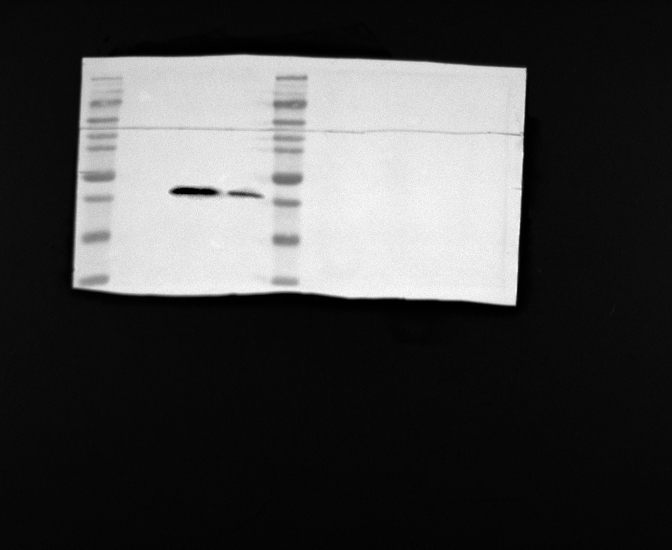

Supplement: Figure 7—source data 2. [file elife-98636-fig7-data2.zip › Figure7_Source Data 2/SCX.jpg]

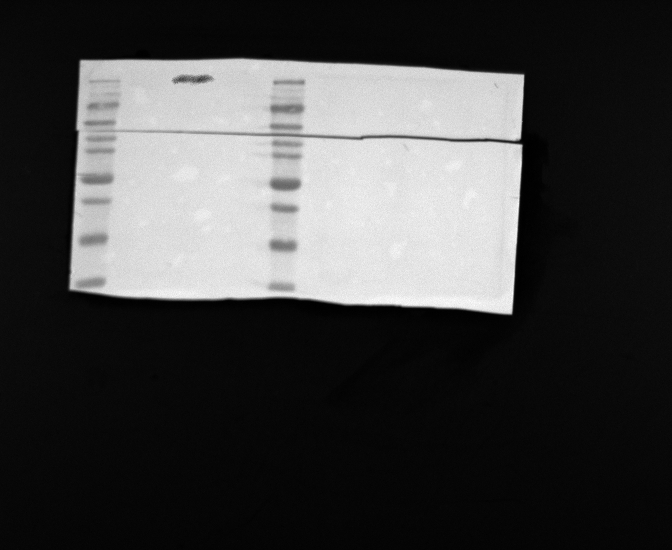

Supplement: Figure 7—source data 2. [file elife-98636-fig7-data2.zip › Figure7_Source Data 2/TNC.jpg]
